# Correlation of blood lipid parameters with newly diagnosed carotid intima media thickness and carotid plaque in primary health care of populations

**DOI:** 10.3389/fcvm.2026.1652451

**Published:** 2026-03-19

**Authors:** Ting Bao, Yanwen Jin, Wei Gao

**Affiliations:** 1 Health Management Center, General Practice Center, West China Hospital, Sichuan University, Chengdu, China; 2 Biliary Surgery, West China Hospital, Sichuan University, Chengdu, China

**Keywords:** atherosclerosis, blood lipids, carotid intima-media thickness (C-IMT), carotid plaque (CP), primary health care

## Abstract

**Introduction:**

Carotid intima-media thickness (CIMT) or carotid plaque has become one of the subclinical markers of atherosclerosis. Different lipids may play different roles in atherosclerosis. However, there are a few studies with a small sample size on the contribution of different lipids to CIMT or carotid plaque, and the conclusions are controversial.

**Methods:**

We included Chinese residents of Chengdu who voluntarily participated in an annual medical check-up at West China Hospital, Sichuan University. Demographic data and medical history of subjects were collected. Anthropometry and laboratory indexes, including blood lipids, were measured. Bilateral carotid arteries were assessed.

**Results:**

A total of 9,356 cases were included in this study. The prevalence of thickened CIMT and carotid plaque were 9.4% and 17.8%, respectively. After adjusting for confounding factors, total cholesterol (TC), low-density lipoprotein cholesterol (LDL-C), nonhigh-density lipoprotein cholesterol, LDL-C/high-density lipoprotein cholesterol (HDL-C) and NHDL-C/HDL-C were independent risk factors for thickened CIMT, but triglyceride (TG), HDL-C and TC/HDL-C had no significant correlation with thickened CIMT. TC, LDL-C,NHDL-C and NHDL-C/HDL-C were independent risk factors for carotid plaque, but TG, HDL-C, LDL-C/HDL-C and TC/HDL-C had no significant correlation with carotid plaque. TC, LDL-C, NHDL-C, LDLC/ HDL-C and NHDL-C/HDL-C were positively correlated with CIMT, while TG, HDL-C, and TC/HDL-C were not.

**Discussion:**

The correlation between different lipid components and thickened CIMT or carotid plaque are different. TC, LDL-C, NHDL-C,LDL-C/HDL-C and NHDL-C/HDL-C were positively correlated with CIMT, but TG, HDL-C and TC/HDL-C not. TC, LDL-C, NHDL-C and NHDL-C/HDL-C were positively correlated with carotid plaque, but TG, HDL-C, LDL-C/HDL-C and TC/HDL-C not.

## Introduction

The incidence of atherosclerotic cardiovascular disease (ASCVD) and stroke is very high and is the main cause of disability and death worldwide (1). Atherosclerosis plays a key role in ASCVD and stroke (2). The location of the carotid artery is superficial, and carotid artery ultrasound is convenient, noninvasive and low-cost. Carotid intima-media thickness (CIMT) and carotid plaque have become subclinical markers of atherosclerosis and predictors of ASCVD and stroke (3). A meta-analysis has shown that CIMT and carotid plaque are closely associated with an increased risk of ASCVD (4). With the understanding of CIMT, it is increasingly being used as a surrogate outcome indicator for atherosclerosis in clinical trials (4–8).

Currently, risk factors for atherosclerosis include male sex, age, smoking, hypertension, diabetes mellitus, smoking, lipid disorders, obesity and so on (9–11). Lipids play a key role in the pathophysiology of arteriosclerosis and are an important modifiable risk factor (12). Different lipid components may play different roles in atherosclerosis. Previous studies have shown that low-density lipoprotein cholesterol (LDL-C) is a risk factor and that high-density lipoprotein cholesterol (HDL-C) is a protective factor against atherosclerosis (13). Some studies have found that nonhigh-density lipoprotein cholesterol (NHDL-C) is a better predictor of atherosclerosis than LDL-C (14). Other studies have found that the ratio of total cholesterol (TC)/HDL-C and LDL-C/HDL-C can better predict atherosclerosis than individual lipid components (15, 16). Some studies have also found that triglycerides (TG) can increase the incidence and mortality of cardiovascular diseases (17, 18).

However, there are a few studies with a relatively small sample size on the contribution of different lipid components to CIMT or carotid plaque, and the conclusions are controversial (19–21). Therefore, it is necessary to study the correlation between different blood lipid parameters and CIMT or carotid plaque through a larger sample size study.

## Materials and methods

### Participants

We included Chinese population who received primary health care in West China Hospital Health Management Center from January 2020 to Decemberr 2021. Subjects with serious cardiovascular and cerebrovascular diseases, severe liver and kidney disease, malignant tumors, and those receiving lipid-lowering drugs were excluded. Patients with a history of carotid artery disease were excluded. This study was approved by the Ethics Committee of West China Hospital, Sichuan University and was performed in accordance with the Declaration of Helsinki. All the participants signed informed consent forms.

### Data collection

Demographic data, smoking and drinking history, family history, and present disease history of subjects were collected. Participants were fasting for at least 8 h. Height, weight, waist circumference, hip circumference, waist-to-hip ratio, blood pressure, fasting plasma glucose (FPG), glycosylated hemoglobin A1c (HbA1c), blood uric acid, and blood lipids were measured. Blood lipids included TG, TC, LDL-C, HDL-C, and NHDL-C. Biochemical indexes were determined by an automatic biochemical analyzer (ROCHE COBASC702, Shanghai), and HbA1c was measured by high-pressure liquid chromatography (TOSOH HLC-723G8, Japan). Fatty liver was assessed by color Doppler ultrasound of the liver (Philips EPIQ7C, America). Body mass index (BMI) = weight/height^2^ (kg/m^2^).

### Carotid artery ultrasound

Bilateral carotid arteries were assessed by an experienced sonographer through a color Doppler ultrasound (Philips EPIQ7C, America). Carotid artery color Doppler ultrasound reports are issued by the operating doctor and reviewed in real time by senior physicians. The probe must be parallel to the vessel wall, and the beam should be perpendicular to the tube wall. The scanning mode of combined longitudinal and transverse sections was adopted. The vertical distance from the upper edge of the inner membrane to the upper edge of the outer membrane in the distal common carotid artery and/or bulb of the carotid artery was defined as CIMT (22). A CIMT less than 1 mm was normal. A 1.0 mm ≤ CIMT < 1.5 mm was defined as thickened CIMT (22). A CIMT ≥1.5 mm, protruding into vascular or localized thickness and 50% higher than peripheral CIMT, was defined as carotid plaque (22). It was discovered by chance during the first-time ultrasound screening.

### Statistical analysis

SAS 9.4 software was used for statistical analysis. The central trend of measurement data is represented by the mean (X). Dispersive trends were expressed by the standard deviation (SD). Qualitative data were described by absolute numbers and rates. The measurement data of two independent samples were compared by t test. The comparison of rates was compared by the chi-square test of four lattice tables. Fisher's exact probability method was used if the test conditions were not satisfied. Multivariate linear regression analysis was used in the multivariate analysis if dependent variables were quantitative variables. Logistic regression was used for multivariate analysis when dependent variables were dichotomous variables. The test level α was 0.05.

## Results

**Figure 1:**
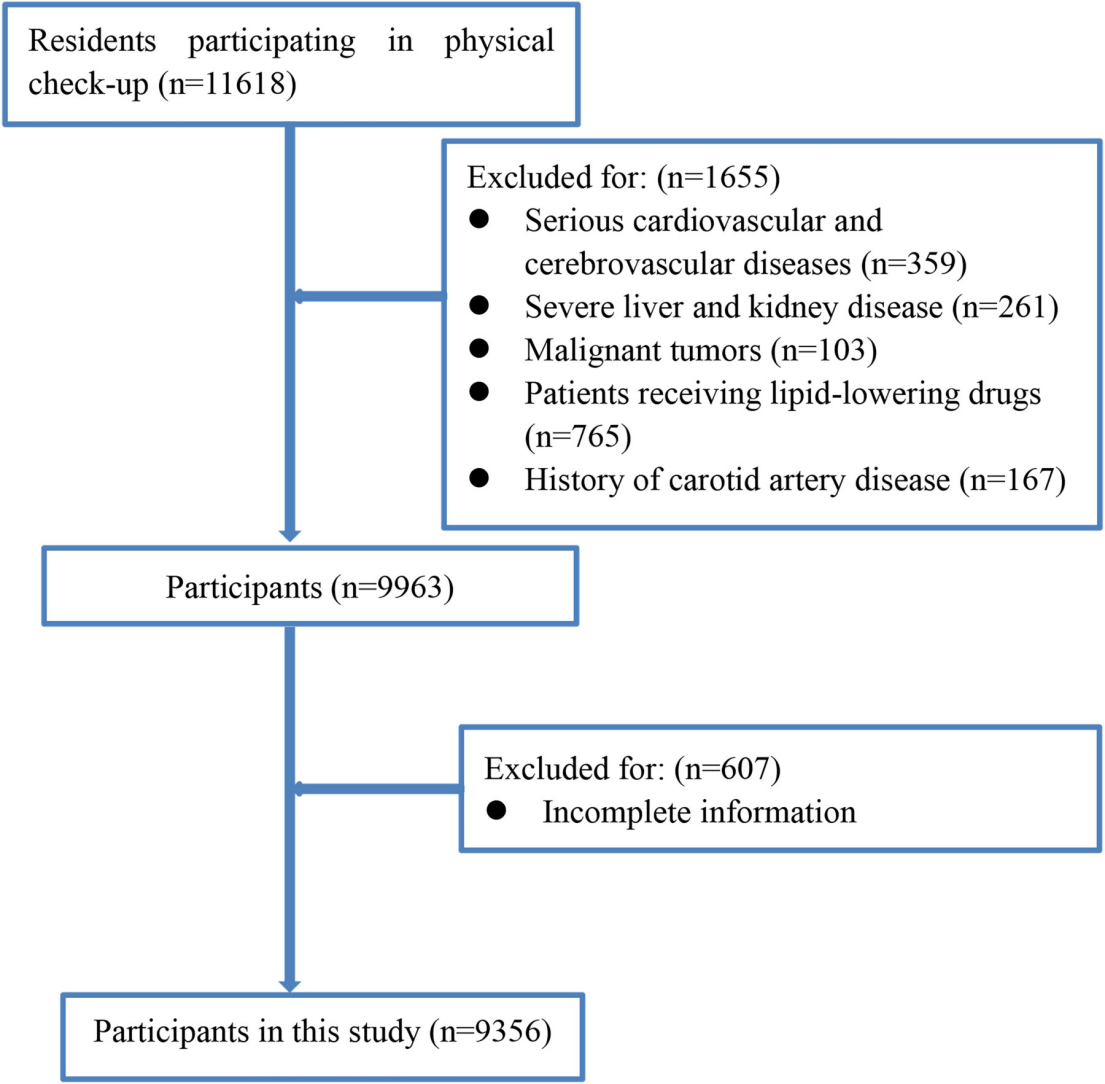
Flowchart of participants enrollment.

From January 2020 to December 2021, a total of 9,356 participants met the inclusion criteria (Figure 1). There were 6,136 males (65.60%) and 3,220 females (34.40%). The mean age was 48.86 ± 14.21 years. Among them, 880 cases (9.40%) had thickened CIMT, and 1,664 cases (17.79%) had carotid plaque. The CIMT was 0.85 ± 0.25 mm. Details are shown in Table 1.

**Table 1:** Baseline characteristics of the participants.

Variables		Results
Gender	Male *n* (%)	6,136 (65.60%)
	Female *n* (%)	3,220 (34.40%)
Age (year)	Mean ± *S*	48.86 ± 14.21
Age group *n* (%)	18–30	760 (8.12%)
	31–40	1,364 (14.58%)
	41–50	2,848 (30.44%)
	51–60	2,412 (25.78%)
	61–70	1,112 (11.89%)
	71–80	496 (5.30%)
	Over 80	364 (3.89%)
Race	Han *n* (%)	9,039 (96.61%)
	Others *n* (%)	317 (3.39%)
Hypertension	*n* (%)	1,295 (13.84%)
Diabetes	*n* (%)	471 (5.03%)
Smoking	*n* (%)	2,483 (26.54%)
Drinking	*n* (%)	843 (9.01%)
Systolic pressure (mmHg)	Mean ± *S*	121.00 ± 16.07
Diastolic pressure (mmHg)	Mean ± *S*	74.48 ± 10.37
Waist circumference (cm)	Mean ± *S*	81.41 ± 10.50
WHR	Mean ± *S*	0.85 ± 0.08
BMI (Kg/m^2^)	Mean ± *S*	23.74 ± 3.24
FPG (mmo/l)	Mean ± *S*	5.29 ± 1.28
HbA1c (%)	Mean ± *S*	5.67 ± 0.78
Blood uric acid (mmo/l)	Mean ± *S*	350.68 ± 87.42
Fatty liver	*n* (%)	2,190 (23.41%)
TG (mmo/l)	Mean ± *S*	1.71 ± 1.30
TC (mmo/l)	Mean ± *S*	4.79 ± 0.90
LDL-C (mmo/l)	Mean ± *S*	2.81 ± 0.78
HDL-C (mmo/l)	Mean ± *S*	1.38 ± 0.39
NHDL-C (mmo/l)	Mean ± *S*	3.42 ± 0.92
TC/HDL-C	Mean ± *S*	3.74 ± 1.22
LDL-C/HDL-C	Mean ± *S*	2.20 ± 0.85
NHDL-C/ HDL-C	Mean ± S	2.74 ± 1.22
Thicken CIMT	*n* (%)	880 (9.40%)
CIMT (mm)	Mean ± *S*	0.85 ± 0.25
Carotid plaque	*n* (%)	1,664 (17.79%)

The proportion of males, age, incidence rate of hypertension or diabetes, rate of smoking, systolic blood pressure, waist circumference, waist-to-hip ratio, BMI, FPG, and HbA1c in the thickened CIMT groupwerehigher than those in the normal carotid artery group. There was no statistically significant difference in each lipid parameter between the two groups (Table 2). Compared with the normal carotid artery group, the proportion of males, age, incidence rate of hypertension or diabetes, rate of smoking, systolic blood pressure, waist circumference, waist-to-hip ratio, BMI, blood uric acid, FPG, HbA1c, and incidence rate of fatty liver ratio were increased in the carotid plaque group. There was also no statistically significant difference in each lipid parameter between the two groups (Table 3). There were no significant differences in other variables except TG between the thickened CIMT group and the carotid plaque group (Table 4).

**Table 2:** Comparison between the thickened CIMT group and the normal carotid artery group.

Variables	Groups		Statistics	*P*-value
	Normal carotid artery group *n* = 6,812	Thicken CIMT group *n* = 880		
Sex				
Male *n* (%)	4,160 (61.07%)	692 (78.64%)	*χ*^2^ = 25.8,182	<0.0001
Female *n* (%)	2,652 (38.93%)	188 (21.36%)		
Age (year)	46.64 ± 13.28	63.23 ± 14.36	*t* = 17.26	<0.0001
Race				
Han *n* (%)	6,580 (96.59%)	868 (98.64%)	*χ*^2^ = 2.6453	0.103
Others *n* (%)	232 (3.41%)	12 (1.36%)		
Hypertension *n* (%)	696 (10.22%)	260 (29.55%)	*χ*^2^ = 66.8743	<0.0001
Diabetes *n* (%)	288 (4.23%)	136 (15.45%)	*χ*^2^ = 47.1478	<0.0001
Smoking rate *n* (%)	1,760 (25.84%)	324 (36.82%)	*χ*^2^ = 11.8946	0.0006
Drinking rate *n* (%)	3,168 (46.51%)	356 (40.45%)	*χ*^2^ = 2.8742	0.090
Systolic pressure (mmHg)	119.3 ± 15.46	129.1 ± 16.76	*t* = 8.61	<0.0001
Diastolic pressure (mmHg)	74.12 ± 10.21	74.01 ± 11.21	*t* = −0.15	0.884
Waist circumference (cm)	80.76 ± 10.39	85.69 ± 10.24	*t* = 6.35	<0.0001
Waist-to-hip ratio	0.85 ± 0.08	0.89 ± 0.07	*t* = 7.25	<0.0001
BMI (Kg/m^2^)	23.60 ± 3.20	24.45 ± 3.28	*t* = 3.52	0.001
FPG (mmol/L)	5.23 ± 1.26	5.84 ± 1.83	*t* = 6.28	<0.0001
HbA1c (%)	5.62 ± 0.76	6.09 ± 1.00	*t* = 7.61	<0.0001
Blood uric acid (mmol/L)	348.1 ± 88.01	354.5 ± 76.24	*t* = 1.02	0.308
Fatty liver *n* (%)	1,532 (22.49%)	240 (27.27%)	*χ*^2^ = 2.5139	0.112
TG (mmol/L)	1.68 ± 1.27	1.53 ± 0.82	*t* = −1.64	0.102
TC (mmol/L)	4.79 ± 0.88	4.80 ± 1.08	*t* = 0.11	0.909
LDL-C (mmol/L)	2.81 ± 0.77	2.87 ± 0.92	*t* = 1.01	0.311
HDL-C (mmol/L)	1.38 ± 0.40	1.36 ± 0.35	*t* = −0.55	0.582
NHDL-C (mmol/L)	3.41 ± 0.90	3.43 ± 1.07	*t* = 0.35	0.729
TC/HDL-C	3.73 ± 1.20	3.72 ± 1.19	*t* = −0.12	0.906
LDL-C/HDL-C	2.20 ± 0.84	2.25 ± 0.95	*t* = 0.78	0.435
NHDL-C/ HDL-C	2.74 ± 1.22	2.72 ± 1.19	*t* = 0.219	0.686

**Table 3:** Comparison between the carotid plaque group and the normal carotid artery group.

Variables	Groups		Statistics	*P*-value
	Normal carotid artery group *n* = 6,812	Carotid plaque group *n* = 1,664		
Sex				
Male *n* (%)	4,160 (61.07%)	1,284 (77.16%)	*χ*^2^ = 24.673	<0.0001
Female *n* (%)	2,652 (38.93%)	380 (22.84%)		
Age (year)	46.64 ± 13.28	63.37 ± 13.68	*t* = 29.36	<0.0001
Race				
Han *n* (%)	6,580 (96.59%)	1,620 (97.36%)	*χ*^2^ = 1.0973	0.294
Others *n* (%)	232 (3.41%)	44 (2.64%)		
Hypertension *n* (%)	696 (10.22%)	588 (35.34%)	*χ*^2^ = 218.163	<0.0001
Diabetes *n* (%)	288 (4.23%)	204 (12.26%)	*χ*^2^ = 68.3892	<0.0001
Smoking rate *n* (%)	1,760 (25.84%)	564 (33.89%)	*χ*^2^ = 15.7148	<0.0001
Drinking rate *n* (%)	3,168 (46.51%)	784 (46.63%)	*χ*^2^ = 0.0771	0.781
Systolic pressure (mmHg)	119.3 ± 15.46	130.9 ± 15.80	*t* = 15.44	<0.0001
Diastolic pressure (mmHg)	74.12 ± 10.21	75.17 ± 11.14	*t* = 1.77	0.076
Waist circumference (cm)	80.76 ± 10.39	85.35 ± 10.14	*t* = 9.03	<0.0001
Waist-to-hip ratio	0.85 ± 0.08	0.89 ± 0.07	*t* = 11.27	<0.0001
BMI (Kg/m^2^)	23.60 ± 3.20	24.40 ± 3.31	*t* = 5.01	<0.0001
FPG (mmol/L)	5.23 ± 1.26	5.70 ± 1.47	*t* = 8.22	<0.0001
HbA1c (%)	5.62 ± 0.76	6.00 ± 0.92	*t* = 10.03	<0.0001
Blood uric acid (mmol/L)	348.1 ± 88.01	361.0 ± 82.36	*t* = 2.81	0.005
Fatty liver *n* (%)	1,532 (22.49%)	472 (28.37%)	*χ*^2^ = 7.9014	0.004
TG (mmol/L)	1.68 ± 1.27	1.76 ± 1.30	*t* = 0.86	0.391
TC (mmol/L)	4.79 ± 0.88	4.80 ± 1.06	*t* = 0.19	0.846
LDL-C (mmol/L)	2.81 ± 0.77	2.78 ± 0.88	*t* = −0.47	0.640
HDL-C (mmol/L)	1.38 ± 0.40	1.36 ± 0.38	*t* = −0.92	0.355
NHDL-C (mmol/L)	3.41 ± 0.90	3.44 ± 1.06	*t* = 0.59	0.555
TC/HDL-C	3.73 ± 1.20	3.76 ± 1.29	*t* = 0.45	0.652
LDL-C/HDL-C	2.20 ± 0.84	2.19 ± 0.93	*t* = 0.03	0.972
NHDL-C/ HDL-C	2.74 ± 1.22	2.76 ± 1.29	*t* = −0.37	0.713

**Table 4:** Comparison between the carotid plaque group and thickened CIMT group.

Variables	Groups		Statistics	*P*-value
	Thicken CIMT group *n* = 880	Carotid plaque group *n* = 1,664		
Sex				
Male *n* (%)	692 (78.64%)	1,284 (77.16%)	*χ*^2^ = 0.1800	0.671
Female *n* (%)	188 (21.36%)	380 (22.84%)		
Age (year)	63.23 ± 14.36	63.37 ± 13.68	*t* = −0.12	0.902
Race				
Han *n* (%)	868 (98.64%)	1,620 (97.36%)	*χ*^2^ = 1.0973	0.399
Others *n* (%)	12 (1.36%)	44 (2.64%)		
Hypertension *n* (%)	260 (29.55%)	588 (35.34%)	*χ*^2^ = 2.1717	0.140
Diabetes *n* (%)	136 (15.45%)	204 (12.26%)	*χ*^2^ = 1.2686	0.260
Smoking rate *n* (%)	324 (36.82%)	564 (33.89%)	*χ*^2^ = 0.5415	0.461
Drinking rate *n* (%)	356 (40.45%)	784 (46.63%)	*χ*^2^ = 2.2254	0.135
Systolic pressure (mmHg)	119.3 ± 15.46	129.1 ± 16.76	*t* = −1.27	0.205
Diastolic pressure (mmHg)	74.12 ± 10.21	74.01 ± 11.21	*t* = −1.22	0.224
Waist circumference (cm)	80.76 ± 10.39	85.69 ± 10.24	*t* = 0.38	0.700
Waist-to-hip ratio	0.85 ± 0.08	0.89 ± 0.07	*t* = −0.20	0.842
BMI (Kg/m^2^)	23.60 ± 3.20	24.45 ± 3.28	*t* = 0.16	0.873
FPG (mmol/L)	5.23 ± 1.26	5.84 ± 1.83	*t* = 1.03	0.301
HbA1c (%)	5.62 ± 0.76	6.09 ± 1.00	*t* = 1.09	0.274
Blood uric acid (mmol/L)	348.1 ± 88.01	354.5 ± 76.24	*t* = −0.96	0.338
Fatty liver *n* (%)	240 (27.27%)	472 (28.37%)	*χ*^2^ = 0.0852	0.770
TG (mmol/L)	1.68 ± 1.27	1.53 ± 0.82	*t* = −2.37	0.018
TC (mmol/L)	4.79 ± 0.88	4.80 ± 1.08	*t* = −0.01	0.990
LDL-C (mmol/L)	2.81 ± 0.77	2.87 ± 0.92	*t* = 1.14	0.253
HDL-C (mmol/L)	1.38 ± 0.40	1.36 ± 0.35	*t* = 0.08	0.932
NHDL-C (mmol/L)	3.41 ± 0.90	3.43 ± 1.07	*t* = −0.04	0.966
TC/HDL-C	3.73 ± 1.20	3.72 ± 1.19	*t* = −0.40	0.687
LDL-C/HDL-C	2.20 ± 0.84	2.25 ± 0.95	*t* = 0.68	0.495
NHDL-C/ HDL-C	2.72 ± 1.19	2.76 ± 1.29	*t* = −0.78	0.558

Logistic regression analysis showed that there was no correlation between lipid parameters and thickened CIMT (*P* > 0.05). We conducted a multicollinearity analysis on the age, sex, history of hypertension, history of diabetes, smoking, drinking, BMI, blood uric acid, fatty liver, and different blood lipids parameters and found that there was multicollinearity BMI,blood uric acid and fatty liver (variance inflation factors was 11.3, 10.5 and 11.2). When uric acid and fatty liver were removed as variables, the inflation factor of BMI was 2.2. In addition, Pearson correlation and point-two column correlation suggested that BMI was correlated with uric acid (*r* = 0.414,*P* = 0.000) or fatty liver (*r* = 0.533,*P* = 0.000). Based on the above two points, we excluded uric acid and fatty liver from the model. After adjusting for age, sex, history of hypertension, history of diabetes, smoking, drinking, BMI, TC [OR = 1.325 (1.132, 1.501)], LDL-C[OR = 1.431(1.215,1.711)],NHDL-C[OR = 1.238(1.145,1.488)], LDL-C/HDL-C[OR = 1.321(1.099, 1.532)] and NHDL-C/HDL-C [OR = 1.298 (1.189, 1.412)] were independent risk factors for thickened CIMT, but TG [OR = 1.125 (0.964,1.249)], HDL-C [OR = 1.238 (0.732,1.855)], and TC/HDL-C [OR = 1.866 (0.909,1.211)] had no significant correlation with thickened CIMT, as shown in Table 5.

**Table 5:** Multivariate logistic regression analysis of thickened CIMT.

Variables	Model 1		Model 2		Model 2	
	OR (95% CI)	*P*-value	OR (95% CI)	*P*-value	OR (95% CI)	*P*-value
TG	0.947 (0.802, 1.018)	0.102	1.087 (0.979,1.207)	0.120	1.125 (0.964,1.249)	0.132
TC	1.010 (0.857,1.174)	0.909	1.211 (1.021, 1.436)	0.028	1.325 (1.132, 1.501)	0.009
LDL-C	1.075 (0.899,1.286)	0.311	1.359 (1.115,1.655)	0.002	1.431 (1.215,1.711)	0.001
HDL-C	0.976 (0.826,1.329)	0.582	1.166 (0.758,1.793)	0.484	1.238 (0.732,1.855)	0.565
NHDL-C	1.057 (0.882, 1.287)	0.729	1.190 (1.018,1.395)	0.039	1.238 (1.145,1.488)	0.021
TC/HDL-C	0.996 (0.826, 1.213)	0.906	1.026 (0.896,1.176)	0.707	1.866 (0.909,1.211)	0.126
LDL-C/HDL-C	1.078 (0.873, 1.287)	0.435	1.222 (1.014,1.472)	0.035	1.321 (1.099,1.532)	0.027
NHDL-C/HDL-C	1.132 (0.911, 1.235)	0.326	1.218 (1.132, 1.353)	0.025	1.298 (1.189, 1.412)	0.019

Model 1: unadjusted.

Model 2: adjusted for age, sex, history of hypertension, history of diabetes, smoking, drinking.

Model 3: adjusted for age, sex, history of hypertension, history of diabetes, smoking, drinking, BMI.

Logistic regression analysis showed that there was no correlation between lipid parameters and carotid plaque (*P* > 0.05). We conducted a multicollinearity analysis on the age, sex, history of hypertension, history of diabetes, smoking, drinking, BMI, blood uric acid, fatty liver, and different blood lipids parameters and found that there was multicollinearity in BMI,blood uric acid and fatty liver (variance inflation factors was 10.2, 11.5 and 10.9). When uric acid and fatty liver were removed as variables, the inflation factor of BMI was 1.9. After adjusting for age, sex, history of hypertension, history of diabetes, smoking, drinking, BMI, TC [OR = 1.346 (1.118,1.543)], LDL-C [OR = 1.202 (1.107,1.432)], NHDL-C [OR = 1.278 (1.109,1.435)] and NHDL-C/HDL-C [OR = 1.187 (1.156, 1.345)] were independent risk factors for carotid plaque, but TG [OR = 1.098 (0.912,1.217) ], HDL-C [OR = 1.231 (0.832,1.589)], TC/HDL-C [OR = 1.132 (0.876,1.332)] and LDL-C/HDL-C [OR = 1.132 (0.955,1.372)] had no significant correlation with carotid plaque after adjusting for confounding factors (Table 6).

**Table 6:** Multivariate logistic regression analysis of carotid plaque.

Variables	Model 1		Model 2		Model 3	
	OR (95% CI)	*P*-value	OR (95% CI)	*P*-value	OR (95% CI)	*P*-value
TG	1.039 (0.864, 1.185)	0.391	1.059 (0.959,1.169)	0.261	1.098 (0.912,1.217)	0.362
TC	1.023 (0.806,1.117)	0.846	1.239 (1.069,1.464)	0.004	1.346 (1.118,1.543)	0.002
LDL-C	0.987 (0.836,1.245)	0.640	1.188 (1.002,1.408)	0.047	1.202 (1.107,1.432)	0.032
HDL-C	0.956 (0.875,1.342)	0.355	1.122 (0.774,1.543)	0.543	1.231 (0.832,1.589)	0.632
NHDL-C	1.121 (0.831, 1.287)	0.555	1.209 (1.046,1.397)	0.010	1.278 (1.109,1.435)	0.009
TC/HDL-C	1.025 (0.920, 1.083)	0.652	1.079 (0.965,1.207)	0.182	1.132 (0.876,1.332)	0.174
LDL-C/HDL-C	1.008 (0.994, 1.087)	0.972	1.105 (0.942,1.297)	0.221	1.132 (0.955,1.372)	0.201
NHDL-C/HDL-C	1.156 (0.834, 1.244)	0.256	1.134 (1.098, 1.366)	0.037	1.187 (1.156, 1.345)	0.029

Model 1: unadjusted.

Model 2: adjusted for age, sex, history of hypertension, history of diabetes, smoking, drinking.

Model 3: adjusted for age, sex, history of hypertension, history of diabetes, smoking, drinking, BMI.

Multiple linear regression analysis indicated that TC, LDL-C, NHDL-C, LDL-C/HDL-C, TC/HDL-C and NHDL-C/HDL-C were positively correlated with CIMT (*P* < 0.05), but TG and HDL-C were not significantly correlated with CIMT (*P* > 0.05). We conducted a multicollinearity analysis on the age, sex, history of hypertension, history of diabetes, smoking, drinking, BMI, blood uric acid, fatty liver, and different blood lipids parameters and found that there was multicollinearity in blood uric acid and fatty liver (variance inflation factors was 10.8, 12.1 and 11.8). When uric acid and fatty liver were removed as variables, the inflation factor of BMI was 1.5. After adjusting for confounding factors, TC, LDL-C, NHDL-C, LDL-C/HDL-C and NHDL-C/HDL-C were positively correlated with CIMT (*P* < 0.05), while TG, HDL-C, and TC/HDL-C were not significantly correlated with CIMT (*P* > 0.05) (Table 7).

**Table 7:** Multivariate linear regression analysis of CIMT and blood lipid parameters.

Variables	Model 1		Model 2		Model 3	
	β (95% CI)	*P*-value	β (95% CI)	*P*-value	β (95% CI)	*P*-value
TG	0.027 (−0.007,0.061)	0.121	0.025 (−0.009,0.060)	0.149	0.029 (−0.010,0.055)	0.165
TC	0.059 (0.034,0.084)	<0.0001	0.047 (0.020,0.075)	0.001	0.050 (0.033,0.086)	0.0005
LDL-C	0.074 (0.046,0.102)	<0.0001	0.062 (0.031,0.094)	<0.0001	0.078 (0.045,1.006)	<0.0001
HDL-C	0.003 (−0.077,0.084)	0.934	0.000 (−0.083,0.084)	0.998	0.007 (−0.078,0.096)	0.985
NHDL-C	0.060 (0.035,0.085)	<0.0001	0.049 (0.021,0.076)	0.001	0.075 (0.045,0.097)	0.0006
TC/HDL-C	0.033 (0.010,0.056)	0.005	0.024 (−0.001,0.048)	0.061	0.035(−0.004,0.076)	0.057
LDL-C/HDL-C	0.048 (0.019,0.077)	0.001	0.035 (0.003,0.066)	0.033	0.045 (0.015,0.082)	0.022
NHDL-C/HDL-C	0.021 (−0.034,0.053)	0.743	0.037 (0.012,0.053)	0.018	0.052 (0.031,0.086)	0.0008

Model 1: unadjusted.

Model 2: adjusted for age, sex, history of hypertension, history of diabetes, smoking, drinking.

Model 3: adjusted for age, sex, history of hypertension, history of diabetes, smoking, drinking, BMI.

## Discussion

### Prevalence rate of thickened CIMT and carotid plaque

Our study found that the prevalence of thickened CIMT and carotid plaque were 9.4% and 17.8%, respectively, in Chengdu residents. Another study, also from China, including 311 community residents, found that the prevalence of thickened CIMT and carotid plaque were 8.4% and 15.8%, respectively, which is similar to the results of our study (19). An epidemiological investigation from Jiangsu Province, China, showed a higher morbidity rate than our study (23). The prevalence of thickened CIMT was 13% in a Japanese study involving a 2012 population aged 34–88 years (24). Differences in prevalence may be related to the different gender compositions, ages, races, diseases, and other potential factors in the included participants. Participants in our study voluntarily underwent a physical check-up, and their literacy, incomes, and health requirements were relatively higher, which may be the main reason for the lower prevalence of thickened CIMT and carotid plaque. The subjects in our research were mainly Han Chinese. Race is an independent predictor of CIMT or carotid plaque in a large multiethnic cohort with a 10-year follow-up (25).

Liu et al. (26) included 3,214 healthy Chinese individuals undergoing physical examinations and found that serum HDL-C, NHDL-C, TC/HDL-C, and LDL/HDL-C were all associated with the incidence of carotid artery plaques. There are both similarities and differences with our research results. Although all the people we included were those undergoing health check-ups, the sample size of our research is larger than that of previous study.our criteria for inclusion and exclusion were different and we excluded patients with a previous history of carotid plaque. All of these might be the reasons for the inconsistent results.

### TG and HDL-C

To date, there has been a controversial correlation between TG or HDL-C and arteriosclerotic disease (17, 18, 20, 21, 27–29) Several studies have suggested that TG is independently associated with an increased risk of coronary artery disease CAD (17, 18). In contrast, a study reported that neither HDL-C nor TG can independently predict carotid plaque (27). A study has even found that TG is a protective factor against carotid plaque (28). The Guidelines of the American College of Cardiology and the European Society of Cardiology do not recommend HDL-C as a target for preventing ASCVD (29).

The controversy about the relationship between TG and arteriosclerosis may be attributed to the complexity of absorption and metabolism of TG and the role of different TG subtypes in the development of atherosclerosis (30). TG can trigger inflammation at the carotid artery intima-media and is then resolved, but cholesterol remains in it to promote foam cell formation and atherosclerosis (31). Assessing the benefits of triglyceride reduction alone is not easy, since many triglyceride-lowering drugs also lower cholesterol.

The concentration of high-density lipoprotein (HDL-P) rather than HDL-C particles is a better predictor of the function of HDL (32, 33). An observational study confirmed a stronger relationship between HDL-P and CAD than HDL-C (34, 35). There is evidence that HDL-P with cholesterol overload may be harmful, as experimental studies have observed that HDL-P with cholesterol overload affects the transport and clearance of cholesterol (36, 37). HDL-P with cholesterol overload is independently associated with the progression of carotid atherosclerosis, which may explain why increased HDL-C is not beneficial. HDL-P and HDL-C together determine antiatherosclerotic function, rather than a single parameter (38). The lipid parameter in our study was HDL-C rather than HDL-P; therefore, no correlation between HDL-C and IMT or carotid plaque was found.

### TC and LDL-C

TC and LDL-C are well-recognized risk factors for atherosclerosis. Studies have shown that TC and LDL-C have the greatest predictive value for thickened CIMT or carotid plaque (19, 39). However, it has been reported that LDL-C and TC are not associated with carotid plaques in populations with a high risk of stroke (21). The researchers explain that the controversy may be due to the high-risk participants included in the study (21). In high-risk populations, the role of lipids may be attenuated (40). The participants in this study were from the general population rather than the high-risk population.

Another interesting finding was that there was no correlation between TC or LDLD-C and carotid artery before adjusting for other confounding factors, but after adjusting for confounding factors, there was a significant correlation between them. Therefore, when conducting multi-factor analysis, it is essential to identify potential confounding factors and make adjustments to prevent the true relationships from being concealed.

### NHDL-C

Most of the studies indicate that the NHDL-C level is a better predictor of IMT and carotid plaque than TC/HDL-C (41–43).

However, Tamada M's study reported that non-HDL-C is not an independent predictor of carotid atherosclerosis (44). The negative results may be attributed to a large number of low-risk subjects in the study, with lower levels of NHDL-C in low-risk subjects than patients with CAD at baseline (44). NHDL-C, which reflects the total particles leading to atherosclerosis, includes very low density lipoprotein cholesterol (VLDL-C), intermedium density lipoprotein cholesterol (IDL-C), and LDL-C and is equal to the TC concentration minus the HDL-C concentration (45). NHDL-C is a good predictive indicator for atherosclerosis.

### TC/HDL and LDL-C/HDL-C ratios

At present, most studies believe that TC/HDL or LDL-C/HDL-C ratios are better predictors of atherosclerosis than TC, LDL-C, or HDL-C alone (15, 16). In contrast, some studies hold controversial points (46). The study has shown that LDL-C/HDL-C is positively associated with CIMT and carotid plaque in male patients with T2DM; however, no correlation has been observed in female patients (45), which may be due to estrogen (47). Our study found that LDL-C/HDL-C was an independent risk factor for thickened CIMT and was positively correlated with CIMT. There was no correlation between TC/HDL-C and the prevalencerate of thickened CIMT or CIMT. There was no correlation between LDL-C/HDL-C or TC/HDL-C and carotid plaque. Possible reasons are as follows. First, LDL-C is more predictive of atherosclerosis than TC (39), so LDL-C/HDL-C is more predictive than TC/HDL-C. Second, HDL-C was not a predictor of atherosclerosis in this study; therefore, the predictive values of LDL-C/HDL-C and TC/HDL-C were reduced. Third, there are differences in risk factors between CIMT and carotid plaque (48). Thickened CIMT and carotid plaque are different stages of the development of atherosclerosis, and their risk factors may be discrepant. Kocaman SA et al. (48) discovered LDL cholesterol, which appear to have a role in later stages of atherosclerosis but not in early stages.

### NHDL-C/HDL-C

Our study found NHDL-C/HDL-C significant correlation between carotid intima-media thickness or carotid plaquein, which was agreement with previous studies. Wang et al. (49) included 27,436 urban workers and among them, 7,161（26.1%） participants were diagnosed with carotid artery plaques. The results show that NHDL-C/HDL-C was related to carotid artery plaques. Liu et al. (50) included 839 subjects at high risk of stroke and found that the non-HDLc/HDLc was positively correlated with the incidence of carotid artery plaques. This study included the high-risk population of cerebral stroke, mainly focusing on non-HDLc/HDLc and carotid plaques. Although the populations included in the three studies were different, the conclusions reached by the three studies were the same. Our research further confirms the previous conclusions.

### Strengths

We included a population that was actively engaged in primary health care, a population that has rarely been reported before. This study included nearly ten thousand participants, while the sample size of previous studies ranged from several hundred to several thousand cases. A larger sample size may produce more reliable results. In this study, patients with newly diagnosed thickened CIMT or carotid plaque screened by physical check-up may benefit from early secondary prevention to prevent ASCVD and stroke. Because our study excluded patients with serious cardiovascular and cerebrovascular diseases, those receiving lipid-lowering drugs and those with a history of carotid artery disease, the conclusion is relatively reliable. In addition, this study analyzed the correlation of various lipid parameters with CIMT, thickened CIMT and carotid plaque, which is relatively comprehensive.

### Limitations

The participants in this study were Chinese residents in Chengdu who voluntarily underwent a physical check-up in a single center, so the results are difficult to generalize to other ethnicities or other regions. Moreover, this study is a cross-sectional study. Although some confounding factors were adjusted, there may still be some potential confounding factors affecting the results. The cross-sectional study could only prove the correlation between blood lipids and CIMT or carotid plaque but not the temporal relationship between lipid parameters and the progression of CIMT or plaque development. So the conclusion we draw is correlation rather than causation. This study did not analyze the relationship between blood lipids and plaque quantity, type, area, thickness, and so on. Carotid artery ultrasound during physical examination is a preliminary screening and does not describe the number and extent of plaques. Carotid ultrasound examinations were performed by experienced physicians and reviewed by senior operators; however, this does not substitute for formal assessment of measurement reproducibility, which might be one of the potential factors influencing the outcome. Furthermore, non-fasting samples, absence of remnant lipoprotein quantification may affect association between TG and HDL-C. Therefore, prospective multicenter studies with higher quality are needed to further confirm causality.

## Conclusions

The correlation between different lipid components and thickened CIMT or carotid plaque are different. TC, LDL-C, NHDL-C and LDL-C/HDL-C were positively correlated with CIMT, but TG, HDL-C and TC/HDL-C not. TC, LDL-C, and NHDL-C were positively correlated with carotid plaque, but TG, HDL-C, LDL-C/HDL-C and TC/HDL-C not.

## Data Availability

The raw data supporting the conclusions of this article will be made available by the authors, without undue reservation.
